# Individual differences in the detection, matching and memory of faces

**DOI:** 10.1186/s41235-018-0111-x

**Published:** 2018-06-27

**Authors:** Matthew C. Fysh

**Affiliations:** School of Psychology, University of Kent, Canterbury, CT2 7NP UK

**Keywords:** Correlation, Face, Detection, Individual differences, Matching, Memory

## Abstract

Previous research has explored relationships between individual performance in the detection, matching and memory of faces, but under limiting conditions. The current study sought to extend previous findings with a different measure of face detection, and a more challenging face matching task, in combination with an established test of face memory. Experiment 1 tested face detection ability under conditions designed to maximise individual differences in accuracy but did not find evidence for relationships between measures. In addition, in Experiments 2 and 3, which utilised response times as the primary performance measure for face detection, but accuracy for face matching and face memory, no correlations were observed between performance on face detection and the other tasks. However, there was a correlation between accuracy in face matching and face memory, consistent with other research. Together, these experiments provide further evidence for a dissociation between face detection, and face matching and face memory, but suggest that these latter tasks share some common mechanisms.

## Significance statement

Despite the abundance of research that has explored face processing abilities such as face detection, face matching and face memory, current understanding of whether these processes might utilise similar perceptual mechanisms is limited. Recent research has begun to address this question and implies that these might comprise independent processes. However, some design limitations, such as the use of accuracy-based measures alone to assess face detection and tests that might suffer from ceiling-level performance, limit the extent to which firm conclusions can be drawn about associations between these processes. In the current study, three experiments are presented that further explore potential relationships between these face-specific tasks, by investigating whether face detection speed and accuracy correlates with face matching and face memory. These latter abilities were assessed using two challenging perceptual tests, namely the Kent Face Matching Test (KFMT) and the long-form version of the Cambridge Face Memory Test (CFMT+). Experiment 1 imitated previous attempts to explore relationships between these processes, by measuring face detection under challenging conditions. In Experiments 2 and 3, potential associations between these tasks were investigated with a greater focus on individual differences in response speed in the detection task, as opposed to response accuracy. The findings suggest that the detection of faces in visual scenes comprises an independent ability, whereas face matching and face memory engage some similar mechanisms. These data hold implications for current understanding of domain specificity in face perception.

## Background

Human face processing consists of a number of distinct but interrelated tasks. The *detection* of faces within the visual environment, for example, enables the subsequent identity *matching* of unfamiliar faces, or the *recognition* of already-known identities. Each of these tasks has been studied in detail (see, e.g., Bindemann & Lewis, 2013; Burton, White, & McNeill, 2010; Johnston & Edmonds, 2009), but little is still known about whether these are conducted by shared or dissociable cognitive mechanisms. In turn, these tasks are characterised by substantial individual differences in performance (see, e.g., Bindemann, Avetisyan, & Rakow, 2012; Bindemann, Brown, Koyas, & Russ, 2012; Robertson, Noyes, Dowsett, Jenkins, & Burton, 2016; Russell, Duchaine, & Nakayama, 2009), but it is unresolved as to whether individuals who are good at face detection are similarly proficient at face matching or face memory. Therefore, the aim of this study is to assess relationships between individual performance in these three tasks.

Studies investigating face detection show that this process is fast and highly accurate under self-paced conditions (e.g., Burton & Bindemann, 2009; Crouzet & Thorpe, 2011; Lewis & Edmonds, 2005). However, detection performance is reduced when changes to the natural width-to-height ratios of faces are made (Pongakkasira & Bindemann, 2015). In contrast, face recognition appears to be remarkably robust to such manipulations (Bindemann, Burton, Leuthold, & Schweinberger, 2008; Hole, George, Eaves, & Rasek, 2002; see also Burton, Schweinberger, Jenkins, & Kaufmann, 2015). Such findings imply that, whilst face detection and face recognition involve the same stimulus category, these are dissociable processes. However, associations between such tasks have also been identified. Face recognition deficits in prosopagnosia, for example, have been linked to orienting failures to faces (see, e.g., Dalrymple, Corrow, Yonas, & Duchaine, 2012; Tsao & Livingstone, 2008), raising the alternative possibility that these tasks might engage similar mechanisms.

This prospect aligns with efforts to establish whether individual differences in performance across different, yet related, face processing tasks can be accounted for by a specific mechanism (see, e.g., Verhallen et al., 2017; Wilhelm et al., 2010). Using a battery of tests (see Herzmann, Danthiir, Schacht, Sommer, & Wilhelm, 2008), Wilhelm et al. (2010) found strong associations between face memory and face perception, and demonstrated that faces are processed independently of objects, implying a face-specific cognitive component. More recently, Verhallen et al. (2017) also found evidence to suggest that performance across four face-processing tasks could be accounted for by a common factor, which they referred to as *f*. This research showed that the ability to *match* unfamiliar faces is strongly associated with unfamiliar face *recognition* but correlates weakly with face *detection*. However, the test of face detection that was employed (the Mooney Face Test; see Mooney, 1956; see also, Verhallen et al., 2014) measures participants’ ability to visually organise black and white shapes into face-like arrangements, rather than assessing the detection of actual human faces. In addition, other research has implied that performance in the Mooney Test dissociates from visual search performance (see Foreman, 1991). This search component is a key element of human face detection, which requires the location of a target within visual scenes (see, e.g., Bindemann & Lewis, 2013; Burton & Bindemann, 2009). From these findings, therefore, it is difficult to establish whether face detection ability is associated with face matching and face memory.

One other recent study also investigated possible relationships between face detection, face matching, and face memory (Robertson, Jenkins, & Burton, 2017). This study identified a correlation between face matching and face memory. However, accuracy in these tasks was not associated with participants’ detection of face-like objects, such as pareidolia faces (Experiment 1) and cloud faces (Experiment 2). A third experiment also found no association between the ability to detect human faces in natural scenes and face matching accuracy, but did not include a measure of face memory.

These findings make intuitive sense, when considering that face matching and face memory both concern the *identification* of face stimuli. The former task requires observers to decide whether one face photograph matches that of another similar but potentially different identity. By contrast, face memory tasks entail a similar identity judgement, but which is based on the extent to which a face image that is stored in memory corresponds to a visual representation of a face that is presented. As a consequence, these tasks should, in theory, overlap to some degree. Indeed, this relationship has been observed repeatedly in previous work (see, e.g., Bobak, Hancock, & Bate, 2016; Burton et al., 2010; Fysh & Bindemann, 2018; Megreya & Burton, 2006; Robertson et al., 2017; Verhallen et al., 2017). By contrast, it seems less intuitive to assert that face detection should be associated with face matching and face memory. This is due to the fact that the detection of a face within a visual display entails a *between*-category distinction to separate faces from non-face objects. On the other hand, face identification entails *within*-category distinctions, to determine whether two similar face images match or mismatch, or whether a face encountered within the visual field matches a facial representation stored in memory.

However, two obstacles arise from the research of Robertson et al. (2017) that limit the extent to which firm conclusions can be drawn about whether face detection is dissociated from the recognition and matching of unfamiliar faces. First, it remains uncertain as to whether face-like objects operate as a reliable proxy for human faces. These objects, which include stimuli such as clouds, may share some characteristics with faces but also exhibit many differences and are, de facto, objects in their own right that are not faces (Churches, Baron-Cohen, & Ring, 2009; Moulson, Balas, Nelson, & Sinha, 2011; Takahashi & Watanabe, 2013). Second, Robertson et al. (2017) only utilised accuracy measures to assess face detection performance. This diverges from earlier studies, which utilised response times when investigating detection performance, given that accuracy is often close to ceiling (see, e.g., Bindemann & Burton, 2009; Bindemann & Lewis, 2013; Burton & Bindemann, 2009). For example, manipulating the orientation of faces to be detected from frontal to profile orientation reduces accuracy slightly from 93% to 89%, but elicits a comparatively large increase in visual search time from 593 ms to 704 ms (Bindemann & Lewis, 2013). In addition, comparisons between people with prosopagnosia and control subjects when detecting faces in visual displays reveal only marginal differences in accuracy, but considerable differences in search time (Garrido, Duchaine, & Nakayama, 2008). Considered together, these studies reflect that proficiency in face detection may be best characterised by response *speed*, as opposed to response *accuracy*, when investigating possible associations between this ability and performance in face matching and face memory tasks.

In light of these observations, the aim of the current study was to further examine relationships between individual performance in the detection, matching and memory of faces. Three tasks were employed for this purpose. The first of these comprised a task in which observers searched complex natural scenes for faces (see Burton & Bindemann, 2009; Pongakkasira & Bindemann, 2015). The second and third tasks comprised challenging tests of face matching and face memory; the Kent Face Matching Test (KFMT; Fysh & Bindemann, 2018) and the long version of the Cambridge Face Memory Test (CFMT+; Russell et al., 2009).

These tests differ from the Glasgow Face Matching Test (GFMT; Burton et al., 2010) and the standard version of the Cambridge Face Memory Test (CFMT; Duchaine & Nakayama, 2006), which were employed by Robertson et al. (2017) and may lack the sensitivity to fully explore the range of individual differences in face matching and face memory. The CFMT, for example, is typically employed as a tool for assessing prosopagnosia (Bobak, Parris, Gregory, Bennetts, & Bate, 2017; Duchaine & Nakayama, 2006; Ulrich et al., 2017), but does not distinguish between individuals at the higher end of the face recognition continuum (Russell et al., 2009). In addition, stimuli in the GFMT comprise two well-lit faces bearing the same pose and expression. Critically, identity-match trials depict the same person photographed minutes apart, thereby presenting the task as a best-case scenario (Burton et al., 2010). By contrast, stimuli in the KFMT comprise one controlled target photograph and a non-controlled image, in which expression, pose and lighting, are unconstrained. In addition, identity matches consist of target photographs that were taken many months apart, resulting in considerable within-person variability. As a consequence, the KFMT provides a more difficult test of face matching than the GFMT (see, Fysh & Bindemann, 2018). Therefore, by replacing the GFMT and CFMT with the KFMT and CFMT+, and by using response time as an additional measure of individual performance in face detection, this study sought to further explore whether correlations exist between face detection, matching and memory performance.

### Experiment 1

In this experiment, observers completed a face detection task, which involved searching for faces within complex natural scenes (see Bindemann & Burton, 2009; Burton & Bindemann, 2009; Pongakkasira & Bindemann, 2015). These scenes were displayed only briefly to maximise individual differences in accuracy. The detection task was followed by the KFMT (Fysh & Bindemann, 2018) and the CFMT+ (Russell et al., 2009). To investigate relationships between these tasks fully, accuracy and the speed with which faces were detected within scenes was explored, and these measures were correlated with face matching and face memory. If the absence of associations between face detection, matching and memory in the study of Robertson et al. (2017) were driven by a lack of sensitivity in the matching (GFMT) and memory (CFMT) tests that were employed, then correlations between performance in these tasks might emerge under these alternative conditions.

## Method

### Participants

Thirty undergraduate Psychology students from the University of Kent (7 men, 23 women) with a mean age of 19.5 years (SD = 1.8) participated in this study in exchange for course credit. All participants reported normal or corrected-to-normal vision. This study was conducted in accordance with the ethical guidelines of the British Psychological Association.

### Stimuli and procedure

#### Task 1: face detection task

**Fig. 1:**
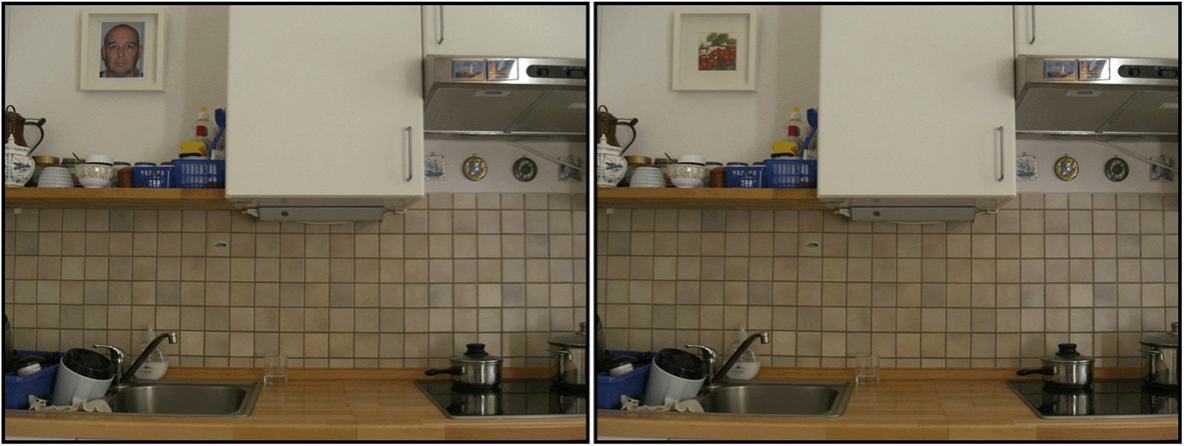
Example stimuli from the face detection task, depicting a target-present (left) and target-absent (right) stimulus display

The face detection task was run using PsychoPy software (Peirce, 2007) and consisted of 100 images of indoor scenes containing a wide range of paraphernalia, such as bookshelves, appliances and furniture (see Burton & Bindemann, 2009). These images were presented at a size of 1024 × 768 pixels with a resolution of 72 ppi. Half of these scenes contained an embedded face photograph, which depicted a Caucasian adult with a neutral expression. The location of faces within scenes varied to ensure that observers had to search for the targets. In addition, the size of the faces varied slightly between images, taking up between 0.08 and 1.73% of the total scene area, to ensure that observers were not utilising a simple search strategy based on the size of faces in each image. Example stimuli are provided in Fig. 1.

At the start of the task, observers were instructed that they would view images of indoor scenes, and that their task was to detect whether or not a face was present within the scene. Responses were provided using a standard computer keyboard. Thus, participants were instructed to press “1” if they located a face within the scene and “2” if they did not. Each trial was preceded by a 1-s fixation cross. This was then replaced by an indoor scene, which was presented on screen for 200 ms, followed by a blank display until a response was registered. Observers were instructed at the beginning of the task that each image would be shown briefly and were asked to respond as quickly and as accurately as possible.

#### Task 2: the Kent Face Matching Test (short form)

**Fig. 2:**
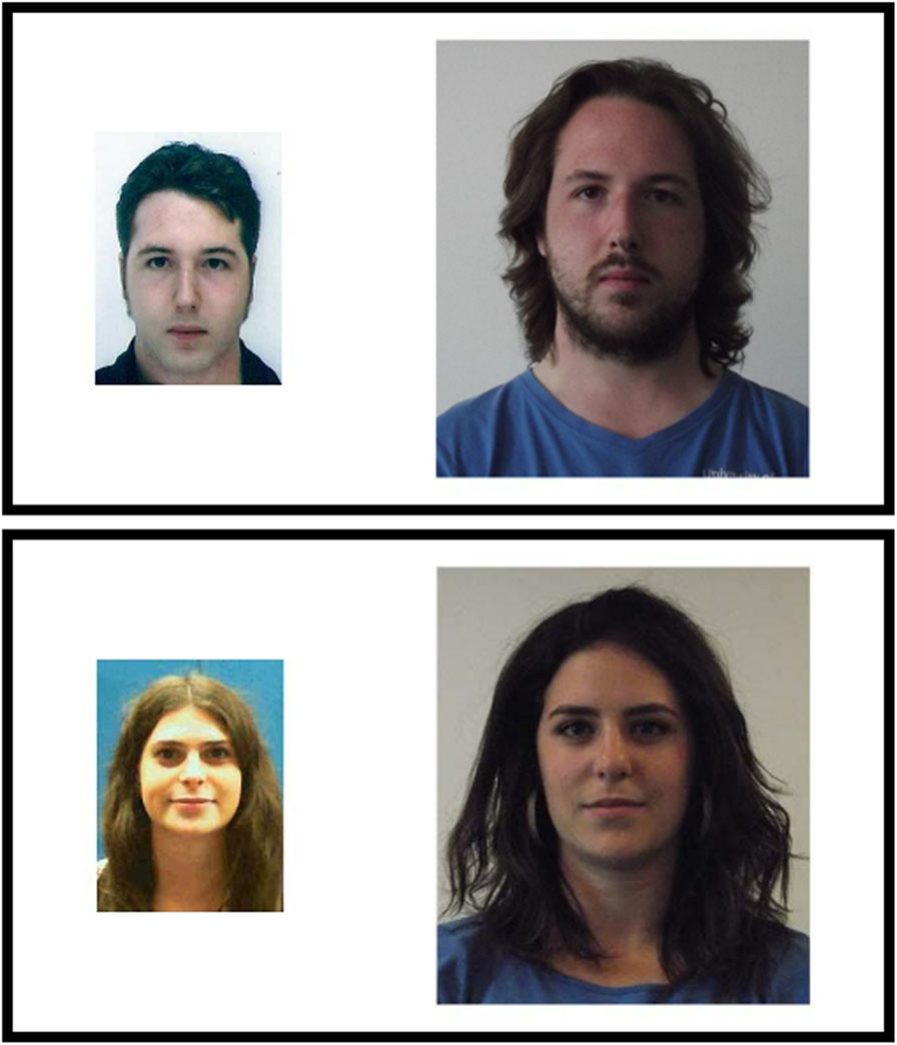
Example stimuli from the Kent Face Matching Test (KFMT; Fysh & Bindemann, 2018). The top pair depicts an identity match, whereas the bottom pair depicts an identity mismatch

Following the face detection task, observers completed the short version of the KFMT, which was also run on PsychoPy. This comprised 40 pairs of Caucasian faces retrieved from the Kent University Face Database (KUFD). Twenty of these stimuli were identity matches, in which both images in a pair depicted the same person. The other 20 face pairs were identity mismatches and depicted two different individuals. Each stimulus category (i.e. matches and mismatches) consisted of 10 male and 10 female subjects. In addition, each pair consisted of a high-quality photograph that was taken under controlled conditions and measured 283 × 332 pixels, and one student ID photograph taken under uncontrolled conditions, measuring 142 × 192 pixels. Full details of the KFMT are provided in Fysh and Bindemann (2018). Example identity pairs are displayed in Fig. 2.

Observers were instructed that their task was to determine whether the pairs of onscreen faces depicted the same person or two different people and were asked to respond as accurately as possible. Response keys “s” and “d” were used to record “same” and “different” responses, respectively. As in the detection task, each trial began with a 1-s fixation cross, which was then replaced by a face pair that remained on screen until a response was registered.

#### Task 3: the Cambridge Face Memory Test+

**Fig. 3:**
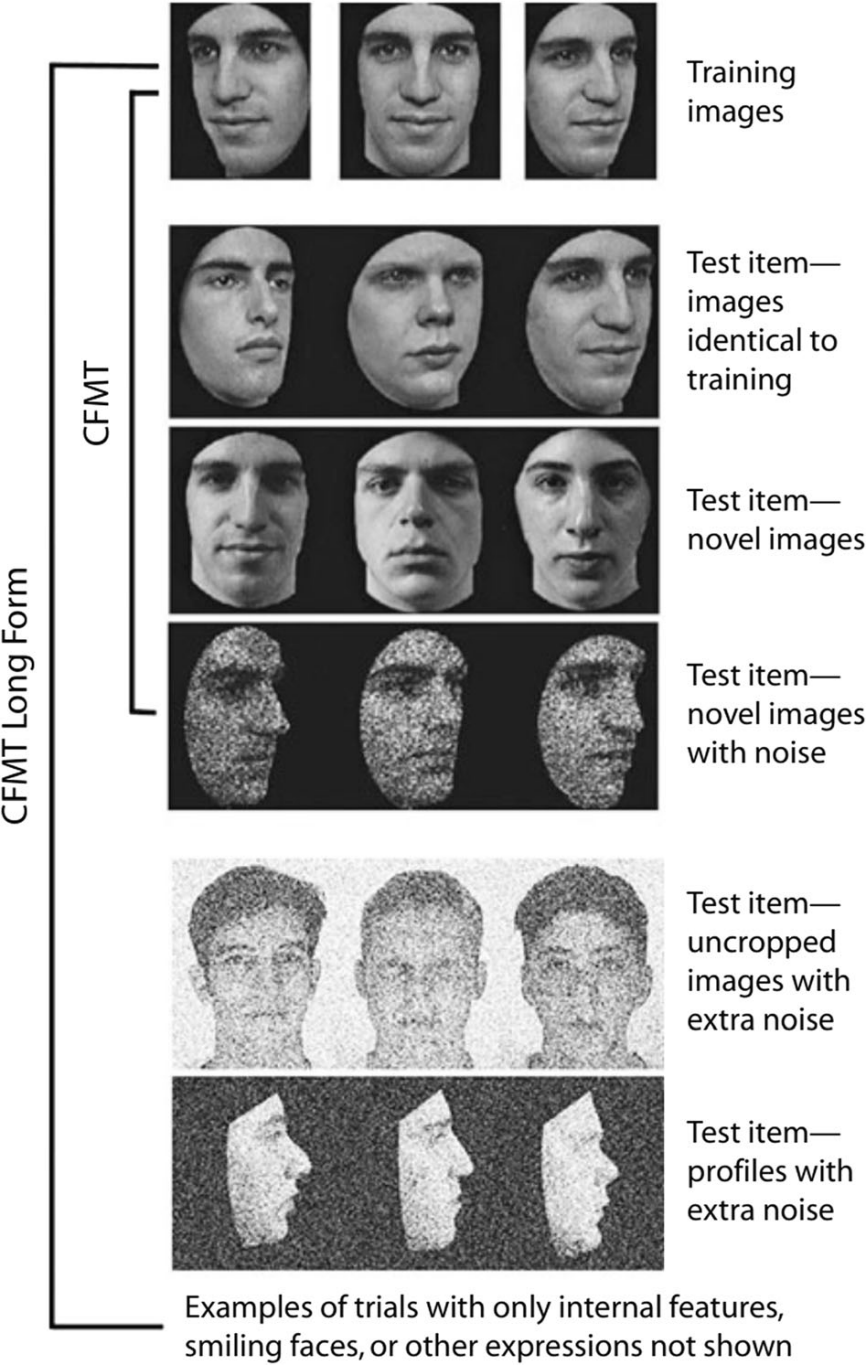
Example stimuli and the structure of the long-form Cambridge Face Memory Test (CFMT+; from Russell et al., 2009)

The final task was the CFMT+ (see, Russell et al., 2009), which was run using Java Script. The CFMT+ consists of 102 trials, of which the first 72 make up the original CFMT (Duchaine & Nakayama, 2006), and an additional block of 30 trials that are considerably more challenging. In the first block, observers studied each individual target depicted across three different viewpoints (frontal, mid-profile right and mid-profile left), and were then asked to identify the target from a three-face array containing two distractor images alongside the studied identity. In the second block, six different but concurrent faces were studied for 20 s. Observers were then presented with a series of three-face arrays containing one target face and two distractor identities and were required to select which face was previously studied. The third block of the task was conceptually similar to the second block, but with the addition of Gaussian noise on top of face images, to further increase the difficulty of the task. In the final block, observers were presented with 30 additional trials that feature heavily degraded face images varying in expression and pose. Example stimuli from each block are displayed in Fig. 3.

## Results

### Summary statistics

For the detection task, mean correct response times were calculated for face-present and face-absent trials. These represent response latency from the moment of stimulus onset and were 0.82 s (SD = 0.23; range 0.51–1.61) and 0.96 s (SD = 0.33; range 0.62–2.02), respectively. Average accuracy for face-present displays was 61% (SD = 15.36%; range 26–90%), and was confirmed to be above chance via a one-sample *t* test, (*t*(29) = 4.07, *p* < 0.001). For face-absent scenes, accuracy was 87% (SD = 6.62%; range 70–98%). For completeness, signal detection measures d’ and criterion were also calculated, which indicate overall performance (sensitivity) and response bias, respectively. Detection sensitivity was 1.48 (SD = 0.47; range 0.53–2.46), and criterion was 0.42 (SD = 0.31; range –0.20–1.35). A one-sample *t* test revealed that criterion was significantly greater than zero, (*t*(29) = 7.37, *p* < 0.001), indicating a response bias to classify displays as not containing a face stimulus.

Next, response times and accuracy-based measures for the KFMT were calculated. Overall mean (M) correct response times were 3.25 s (SD = 1.59; range 1.18–8.12), with comparable response times for match (M = 3.22 s, SD = 1.79; range 1.19–7.55) and mismatch trials (M = 3.27 s, SD = 1.53; range 1.17–8.56). Overall accuracy was 66% (SD = 8.08%; range 52–80%), and 63% (SD = 12.08%; range 40–90%) for match and 69% (SD = 12.46%; range 45–95%) for mismatch trials. In addition, signal detection scores d’ and criterion were 0.89 (SD = 0.48; range 0.13–1.68) and 0.10 (SD = 0.29; range –0.58–0.82), respectively. A one-sample *t* test revealed that criterion was comparable to zero, *t*(29) = 1.84, *p* = 0.077.

Finally, mean accuracy on the CFMT+ across all blocks was 66% (SD = 9.86%; range 47–88%). Accuracy in Block 1 was 99% (SD = 1.92%; range 94–100%), which declined subsequently over the second (M = 77%; SD = 13.50%; range 47–100%), third (M = 61%; SD = 22.06%; range 17–96%), and final block (M = 39%; SD = 10.95%; range 13–63%).

### Correlations

To investigate whether these tasks are associated, a series of correlational analyses were performed. Considering that face detection is characterised typically by response speed (Bindemann & Lewis, 2013) but matching and memory performance by accuracy (Fysh & Bindemann, 2018; Russell et al., 2009), this analysis correlated response times and accuracy measures separately but also investigated combinations of these. Note that these were not conducted for target-absent trials of the detection task, as no faces were shown in this condition. Uncorrected results are subsequently reported, with a significance threshold of 0.05. However, in line with recent work (see McCaffery, Robertson, Young, & Burton, in press), the Benjamini-Hochberg adjustment was also implemented with a false discovery rate of 0.20, to correct for multiple correlations (see Benjamini & Hochberg, 1995).

#### Accuracy correlations

Percentage accuracy in the detection task on face-present trials did not correlate with overall accuracy in the KFMT (*r*(28) = 0.134, uncorrected *p* = 0.480), nor with accuracy on match (*r*(28) = 0.281, uncorrected *p* = 0.133) or mismatch trials (*r*(28) = − 0.098, uncorrected *p* = 0.606). Percentage accuracy on face-present trials was associated with performance in the CFMT+ (*r*(28) = 0.431, uncorrected *p* = 0.017) but this was not significant after the Benjamini-Hochberg adjustment for multiple comparisons. Sensitivity in the detection task did not correlate with d’ in the KFMT (*r*(28) = − 0.158, uncorrected *p* = 0.404) or with the CFMT+ (*r*(28) = 0.249, uncorrected *p* = 0.185).

Percentage accuracy in the KFMT was not associated with the CFMT+ (*r*(28) = 0.267, uncorrected *p* = 0.154) nor were its match (*r*(28) = 0.222, uncorrected *p* = 0.239) and mismatch subcomponents (*r*(28) = 0.131, uncorrected *p* = 0.489). In addition, face matching sensitivity did not correlate with face memory performance, (*r*(28) = 0.258, uncorrected *p* = 0.168).

#### Latency correlations

Next, correlations were tested between the mean correct response times in the detection and matching tasks. Note that the CFMT+ does not provide such data, hence it is not included in this analysis. Response times to faces in the detection task did not correlate with overall response times in the KFMT (*r*(28) = 0.323, uncorrected *p* = 0.081) or for the match and mismatch subcomponents (*r*(28) = 0.336, uncorrected *p* = 0.070 and *r*(28) = 0.280, uncorrected *p* = 0.134, respectively).

#### Combinations of accuracy and response latency

Finally, possible associations between response time and accuracy measures were investigated on the basis that the former is typically employed as the primary measure to characterise face detection in natural scenes, but accuracy is the primary measure in matching and memory tasks. Mean correct response times in the detection task for face-present trials correlated with overall accuracy on the KFMT (*r*(28) = 0.394, uncorrected *p* = 0.031). However, this association was not significant following the Benjamini-Hochberg correction. Response speed in the detection task did not correlate with accuracy on match and mismatch trials (*r*(28) = 0.212, uncorrected *p* = 0.260 and *r*(28) = 0.305, uncorrected *p* = 0.101, respectively). In addition, the association between response latency in the detection task and face-matching sensitivity was approaching statistical significance (*r*(28) = 0.357, uncorrected *p* = 0.053), but was not significant after the Benjamini-Hochberg adjustment. Finally, face-detection latency did not correlate with accuracy on the CFMT+ (*r*(28) = − 0.186, uncorrected *p* = 0.324).

## Discussion

This experiment investigated associations in individual performance in the detection, matching and memory of human faces. Accuracy in the detection task was 61% and 87% for target-present and target-absent displays, respectively. These accuracy rates are substantially lower than those reported in detection studies employing unconstrained viewing times but are similar to those reported by Robertson et al. (2017), suggesting that the large number of errors could be attributed to the brief display duration of visual scenes. Overall accuracy in the KFMT was 66%, which aligns with established performance in this test (see, Fysh & Bindemann, 2018). Likewise, overall accuracy was 66% for the CFMT+.

The data revealed some moderate-sized correlations, such as between detection accuracy and performance on the CFMT+ and between detection response times and accuracy on the KFMT. However, these associations did not remain significant following the Benjamini-Hochberg adjustment. In addition, there was no association between accuracy in detection and the KFMT or between the KFMT and CFMT+, and detection response times did not correlate with accuracy in the CFMT+.

Together, these results suggest that the detection, matching and memory of faces comprise separate tasks that engage different mechanisms. However, due to some aspects of the current design, it is difficult to draw any firm conclusions from these data. For example, a response bias was observed in the detection task whereby participants erroneously classified a large number of displays as not containing a face stimulus. It is possible that this bias emerged due to the highly constrained viewing times that were employed (of 200 ms), which limited observers’ ability to make eye movements around visual displays (see, e.g., Henderson, 2003; Rayner, 1998). Such eye movements are necessary to search visual displays for faces and it is this search component that appears to distinguish detection from other tasks with faces (Bindemann & Lewis, 2013). As a consequence, the possibility remains that face detection might correlate with face matching and face memory, but under a different set of conditions that provide observers with unlimited viewing time of stimulus displays. This modification is likely to produce very high accuracy but should also serve to amplify individual differences in response times, which have been previously used to measure detection performance (see, e.g., Bindemann & Lewis, 2013). Therefore, if there is an association between face detection, face matching and face memory, then this may be best characterised by a correlation between response *speed* in the detection task and response *accuracy* in the matching and memory tasks. This was investigated in Experiment 2.

### Experiment 2

In this experiment, observers completed a self-paced face detection task, followed by the KFMT (Fysh & Bindemann, 2018) and the CFMT+ (Russell et al., 2009). As in Experiment 1, the accuracy and speed with which faces are detected within scenes was explored and correlated with face matching and face memory performance. If associations exist between face detection, face matching and face memory, then these might emerge in the form of correlations between the accuracy or speed with which faces are located in visual scenes and accuracy in the matching and memory task.

## Method

### Participants, stimuli and procedure

Thirty new undergraduate Psychology students from the University of Kent (5 men, 25 women) with a mean age of 19.5 years (SD = 3.0) participated in this study in exchange for course credit. All participants reported normal or corrected-to-normal vision. The stimuli and procedure in this experiment were identical to Experiment 1, except that the stimuli of the detection task now remained on screen until a response was registered, and observers were instructed to respond with number key “1” if they located a face within a scene and “2” if a face was not present.

## Results

### Summary statistics

Once again, accuracy and response times for the detection task were calculated first. Observers were faster to detect faces within scenes (M = 2.53 s, SD = 1.02; range 1.60–5.23) than they were to terminate a search when a face was not present (M = 4.40 s, SD = 2.04; range 1.78–11.44). In addition, accuracy was high for both target-present (M = 94%, SD = 7.56%; range 70–100%) and target-absent displays (M = 94%, SD = 12.38%; range 34–100%). Signal detection scores d’ and criterion were 3.19 (SD = 0.77; range 0.11–4.11), and 0.02 (SD = 0.22; range –0.47–0.49), respectively. A one-sample *t* test revealed that criterion was comparable to zero (*t*(29) = 0.42, *p* = 0.676), indicating the absence of a response bias in this task.

For the KFMT, overall mean correct response times were 4.76 s (SD = 3.93; range 1.17–20.76), with longer response times on match (M = 5.49 s, SD = 6.30; range 0.99–30.27) compared to mismatch trials (M = 4.55 s, SD = 3.37; range 1.30–17.85). Overall accuracy was 68% (SD = 8.27%; range 52–90%) and was slightly higher on mismatch trials (M = 70%, SD = 15.08%; range 45–100%) compared to match trials (M = 66%, SD = 14.15%; range 25–95%). Sensitivity was 1.04 (SD = 0.55; range 0.13–2.68). In addition, criterion was 0.08 (*SD* = 0.38; range –0.76–1.16) and was comparable to zero (*t*(29) = 1.13, *p* = 0.269).

Accuracy on the CFMT+ was 70% (SD = 13.13%; range 48–93%). In the first block, accuracy was at 99% (SD = 1.92%; range 94–100%), which declined to 78% (SD = 20.93%; range 30–100%), 69% (SD = 17.39%; range 29–100%) and 44% (SD = 15.57%; range 23–77%) in Blocks 2, 3 and 4, respectively.

### Correlations

As in Experiment 1, correlations were tested separately for response times and accuracy but combinations of these measures were also explored. Again, uncorrected results are reported here with a significance threshold of 0.05, but the Benjamini-Hochberg adjustment was also implemented to correct for multiple comparisons.

#### Accuracy correlations

Correlational analyses did not reveal a relationship between accuracy on face-present trials of the detection task and overall accuracy on the KFMT (*r*(28) = − 0.090, uncorrected *p* = 0.638) or with match (*r*(28) = 0.222, uncorrected *p* = 0.239) and mismatch trials (*r*(28) = − 0.306, uncorrected *p* = 0.100). Similarly, detection sensitivity was not correlated with d’ on the KFMT (*r*(28) = − 0.106, uncorrected *p* = 0.578). In addition, neither accuracy nor sensitivity in the face detection task correlated with accuracy on the CFMT+ (*r*(28) = − 0.003, uncorrected *p* = 0.988 and *r*(28) = − 0.021, uncorrected *p* = 0.914, respectively).

By contrast, there was a positive correlation between performance on the CFMT+ and overall accuracy on the KFMT (*r*(28) = 0.491, uncorrected *p* = 0.006), and accuracy on match trials (*r*(28) = 0.365, uncorrected *p* = 0.047). Both of these associations remained significant following the Benjamini-Hochberg adjustment. However, accuracy on mismatch trials was not associated with performance on the CFMT+ (*r*(28) = 0.196, uncorrected *p* = 0.299). Finally, there was a positive correlation between d’ in the KFMT and performance in the CFMT+ (*r*(28) = 0.479, uncorrected *p* = 0.007). This remained significant following the Benjamini-Hochberg adjustment.

#### Latency correlations

Next, correlation analyses were performed between the mean correct response times in the detection and matching tasks. Once again, the CFMT+ is not included in this analysis as it does not provide such data. Response times to faces in the detection task correlated positively with overall response times in the KFMT (*r*(28) = 0.386, uncorrected *p* = 0.035) and with response times on mismatch trials (*r*(28) = 0.420, uncorrected *p* = 0.021). Both of these associations remained significant following the Benjamini-Hochberg adjustment. There was no association between detection speed and response times on match trials (*r*(28) = 0.286, uncorrected *p* = 0.125).

#### Combinations of accuracy and latency

The final analyses combined response time as a measure of face detection and accuracy as a measure of face matching and memory in the correlational analysis. Response times in the face detection task did not correlate with overall accuracy or sensitivity on the KFMT (*r*(28) = 0.133, uncorrected *p* = 0.485 and *r*(28) = 0.113, uncorrected *p* = 0.552, respectively). Face detection latency also did not correlate with accuracy on match and mismatch trials (*r*(28) = 0.032, uncorrected *p* = 0.869 and *r*(28) = 0.116, uncorrected *p* = 0.542, respectively) or with accuracy on the CFMT+ (*r*(28) = 0.097, uncorrected *p* = 0.609).

## Discussion

This experiment further investigated associations in individual ability to detect, match and recognise faces, but with a detection task that permitted observers to make eye movements when searching visual displays. Performance in this task was notably higher than in Experiment 1 and was near-ceiling for both target-present (94%) and target-absent (94%) displays. In addition, response times were considerably longer on face-present displays in this experiment compared to Experiment 1, reflecting that observers were utilising the time to search displays for face targets. Unlike in Experiment 1, however, there was no evidence of a response bias in the detection task, raising the possibility that the tendency to classify scenes as “target-absent” in the previous experiment emerged due to insufficient time to locate a face stimulus within the visual scene. Overall accuracy in both the KFMT and CFMT+ was comparable to Experiment 1 and was 68% and 70%, respectively.

In terms of accuracy, face detection did not correlate with face matching and face memory. On the other hand, response speed in the detection task correlated with overall response times in the KFMT, and with mismatch, but not match trials. However, these results should be interpreted cautiously as evidence that these tasks engage similar mechanisms, and they may instead simply reflect observers’ capacity for responding quickly. More importantly, the primary aim of this experiment was to ascertain whether there is an association between combinations of response speed in the face detection task and accuracy in the matching and memory task. No such associations were found, providing further evidence that face detection is dissociated from face matching and face memory.

There was a correlation between face memory performance on the CFMT+ and overall accuracy on the KFMT. This differs from Experiment 1, but converges with previous studies that also identified associations between face matching and face memory performance (see, e.g., Fysh & Bindemann, 2018; Robertson et al., 2017; Verhallen et al., 2017). Although the reasons for this correlation only being identified in one of these experiments are not clear-cut; one possible explanation is that this is due to the small sample sizes used. Small samples, such as those in the current experiments, can generate unstable measures of correlation (Schönbrodt & Perugini, 2013). In addition, the range in performance on the KFMT was much greater in Experiment 2 (52-90%) than in Experiment 1 (52–80%), which may also be attributable to the limited number of participants. To validate these results, therefore, a final experiment was conducted, which was identical to Experiment 2 but featured a substantially larger sample of participants.

### Experiment 3

Experiment 2 investigated whether face detection performance, as characterised by both response speed and response accuracy, was associated with proficiency for matching and remembering faces. The results did not imply that performance in face detection is associated with face matching and face memory accuracy, although these latter abilities correlated. However, these findings were obtained from a small sample of 30 observers. In addition, some correlations were moderate in size but were not statistically significant, implying a lack of statistical power. The purpose of this experiment therefore was to clarify the findings of Experiment 2 with a larger sample size.

## Method

### Participants, stimuli and procedure

Seventy new participants (10 men, 60 women) with a mean age of 20 years (SD = 1.91) studying at the University of Kent were recruited for this experiment. None had taken part in the previous experiments, and all reported normal (or corrected-to-normal) vision. The stimuli and procedure were identical to those of Experiment 2.

## Results

### Summary statistics

For the detection task, correct responses were faster on face-present trials (M = 1.11 s; SD = 0.30; range 0.70–2.05) than on face-absent trials (M = 2.82 s; SD = 1.57; range 1.00–7.88). Mean accuracy for face-present trials was 95% (SD = 5.74%; range = 72–100%) and was 98% (SD = 2.73%; range 88–100%) on face-absent trials. In addition, sensitivity was 3.34 (SD = 0.39; range 2.14–4.11) and criterion value was 0.05 (SD = 0.21; range –0.39–0.64). A one-sample *t* test indicated that this was above zero (*t*(69) = 2.12, *p* = 0.038), implying a slight bias to classify scenes as not containing a face stimulus.

For the KFMT, overall mean correct response times were 3.07 s (SD = 1.47; range 1.37–8.66). Accurate responses were faster on match trials (M = 2.86 s; SD = 1.43; range 1.22–7.63) compared to mismatch trials (M = 3.39 s; SD = 1.79; range 1.33–9.87). Overall accuracy was 68% (SD = 9.53%; range 48–90%), and was slightly higher on match (M = 68%; SD = 15.30%; range 30–100%) versus mismatch trials (M = 67%; SD = 16.01%; range 30–100%). Sensitivity and criterion were 1.02 (SD = 0.59; range –0.13–2.68) and − 0.01 (SD = 0.39; range –0.76–1.02), respectively. Criterion was comparable to zero (*t*(69) = − 0.32, *p* = 0.749).

Finally, overall performance in the CFMT+ was 66% (*SD* = 9.99%; range 46–93%), with 98% accuracy (SD = 3.92%; range 83–100%) in Block 1. This declined to 77% (SD = 14.82%; range 40–100%) in the second block, 62% (SD = 18.67%; range 17–100%) in the third block and 40% (SD = 10.49%; range 13–77%) in the final block.

### Correlations

As in the previous experiments, a series of correlational analyses were performed on these data to investigate possible associations between tasks based on accuracy, sensitivity and mean correct response times. For the detection task these were based on face-present trials only. Again, uncorrected results are reported with a significance threshold of 0.05, and significant associations were followed up using the Benjamini-Hochberg adjustment for multiple comparisons.

#### Accuracy correlations

Accuracy in the detection task did not correlate with overall accuracy in the KFMT (*r*(68) = − 0.003, uncorrected *p* = 0.979) or with accuracy on match and mismatch trials (*r*(68) = − 0.036, uncorrected *p* = 0.764 and *r*(68) = 0.031, uncorrected *p* = 0.798, respectively). In addition, d’ did not correlate between tasks (*r*(68) = − 0.086, uncorrected *p* = 0.480). Neither accuracy on face-present trials nor detection sensitivity correlated with performance on the CFMT+ (*r*(68) = 0.188, uncorrected *p* = 0.119 and *r*(68) = − 0.097, *p* = 0.425, respectively).

Overall accuracy and sensitivity in the KFMT correlated significantly with the CFMT+ (*r*(68) = 0.313, uncorrected *p* = 0.008 and *r*(68) = 0.299, uncorrected *p* = 0.012, respectively). Both of these relationships remained significant following the Benjamini-Hochberg correction. However, performance in the CFMT+ was not associated with accuracy on match (*r*(68) = 0.214, uncorrected *p* = 0.075) or mismatch trials (*r*(68) = 0.167, uncorrected *p* = 0.166).

#### Latency correlations

Response latency for face-present trials in the detection task did not correlate with overall response times in the KFMT (*r*(68) = 0.224, uncorrected *p* = 0.062) nor with response times on match trials (*r*(68) = 0.101, uncorrected *p* = 0.404). However, detection speed correlated with response times on mismatch trials (*r*(68) = 0.286, uncorrected *p* = 0.016) and remained significant following the Benjamini-Hochberg correction.

#### Combinations of accuracy and latency

Response latency in the detection task was not associated with overall accuracy or sensitivity in the KFMT (*r*(68) = − 0.101, uncorrected *p* = 0.405 and *r*(68) = − 0.104, uncorrected *p* = 0.392, respectively). In addition, detection response times did not correlate with accuracy on the match and mismatch subcomponents of the KFMT (*r*(68) = 0.111, uncorrected *p* = 0.359 and *r*(68) = − 0.227, uncorrected *p* = 0.059, respectively). Finally, response times in the face detection task were not associated with performance in the CFMT+ (*r*(68) = − 0.154, uncorrected *p* = 0.203).^1^

## Discussion

This experiment sought to replicate the findings of Experiment 2 using a larger sample size. A response bias to classify scenes as face-absent was observed in the detection task. However, accuracy for both face-present and face-absent displays was nonetheless close-to-ceiling at 95% and 98%, respectively.

As in the previous experiments, no accuracy-based associations were identified between face detection and face matching and memory. Response speed in the detection task did not correlate with accuracy in the KFMT and CFMT+ but did correlate with mismatch response times in the KFMT. As in Experiment 2, this latter association should again be interpreted cautiously as evidence for an association between face detection and face matching, given the absence of a correlation between detection speed and matching accuracy. On the other hand, there was a correlation between face matching and face memory accuracy. This finding aligns with the previous experiment and with several recent studies (e.g., Fysh & Bindemann, 2018; Robertson et al., 2017; Verhallen et al., 2017). Together, these results converge with those of Robertson et al. (2017) to imply that whilst face detection functions independently to face matching and face memory, these latter abilities may engage similar mechanisms.

## General discussion

This study explored relationships in individual performance between the detection, matching and memory of faces. In Experiment 1, face-detection performance was measured under a set of conditions designed to amplify individual differences in accuracy, but which prevented observers from searching visual displays (see Robertson et al., 2017). Under these conditions, face-detection accuracy did not correlate significantly with face matching and face memory, although some correlations were moderate in size. Experiments 2 and 3 explored whether associations exist between these tasks when using a detection task that allows observers to make eye movements around visual displays (see, e.g., Bindemann & Lewis, 2013), emphasising response times as a performance measure. The purpose of this was to investigate the possibility that the time taken to locate a face within a display might correlate with accuracy for face matching and face memory. However, there was no correlation between response speed in the detection task and response accuracy in the matching and memory tasks. Together, these experiments suggest that face detection functions independently of face matching and face memory, and instead engages a separate, more specific mechanism.

These experiments also found mixed evidence for an accuracy-based relationship between face matching and face memory. Previous research has demonstrated moderate-to-strong associations between these tasks (see, e.g., Bobak, Hancock, & Bate, 2016; Fysh & Bindemann, 2018; Robertson et al., 2017; Verhallen et al., 2017). This association was not identified in Experiment 1, but emerged in Experiments 2 and 3. Although it is not immediately clear as to why this relationship was not also evident in Experiment 1, it is worth noting the more restricted range in performance in this experiment on the KFMT. It is therefore possible that, perhaps due to its limited number of participants, Experiment 1 did not adequately capture the full range of individual performance on this task. By contrast, the ranges in performance observed in Experiments 2 and 3 were larger. Conversely, these experiments also identified moderate correlations between the face matching and face memory tasks of 0.49 and 0.31, respectively. These are comparable in size to correlations between face matching and memory reported in other recent studies (see, Fysh & Bindemann, 2018; Robertson et al., 2017; Verhallen et al., 2017). Together, these experiments could be interpreted as reflecting that face matching and face memory sometimes engage similar processes, but also rely on additional, unrelated processes.

The consistent finding across Experiments 1–3 that face detection is dissociated from face matching and face memory aligns with the results of Robertson et al. (2017). The former study demonstrated that the detection of human faces was not related to accuracy in a matching task for which performance is typically high, at around 80% (see, e.g., Burton et al., 2010). The current research complements this work by further demonstrating that face detection is dissociated from face matching with a more demanding matching task, for which accuracy is around 66% (Fysh & Bindemann, 2018). In addition, the present study also extends the findings of Robertson et al. (2017) by demonstrating that the detection of human faces is dissociated from face memory performance. Considered together, both of these studies consistently show that face detection operates separately to face matching and face memory. Further, the findings of the current study add that high proficiency in face detection does not imply high proficiency in face identification.

The current work also offers some further insight into the possibility of specific face-processing ability, such as Verhallen’s *f* (Verhallen et al., 2017). In their recent study, Verhallen et al. (2017) found that performance in the Mooney Test, which entails perception of a face from black and white shapes, was associated with face matching and face memory ability. However, although the Mooney Test was described by the researchers as a test of face detection, this task differs importantly from face detection as measured in the current study. Specifically, the Mooney Test measures observers’ ability to organise black and white shapes into a face-like arrangement, as opposed to visually searching for a realistic representation of a human face within complex natural scenes. Moreover, these Mooney arrangements are not faces *per se*, but rather represent face-like objects, and can therefore be of only limited value to understanding how human faces are processed. The current results suggest, therefore, that whilst Verhallen’s *f* may account for shared variability in face memory and face matching, this does not underpin the ability to detect actual faces.

This interpretation is consistent with research demonstrating clinical dissociations between these tasks, such as that impaired face recognition does not necessarily converge with impaired face detection. For example, whilst some prosopagnosic observers exhibit impairment in both face detection and face identification, others perform poorly on identity processing tasks but comparably to controls on detection tasks (Dalrymple & Duchaine, 2016), thereby indicating that these constitute independent abilities. This makes some sense when considering that face detection depends on the ability to make between-category discriminations, in order to reliably distinguish a face from other objects. By contrast, face identification depends on within-category discrimination, given that all faces share a common template, but are nonetheless unique in terms of identity. In line with this reasoning, it has also been argued that face detection should, in fact, be dissociated from identification, on the basis that a good face detector should constitute a poor face identifier (Tsao & Livingstone, 2008).

Converging with established work (see, e.g., Burton et al., 2010; Hancock, Bruce, & Burton, 2000; Johnston & Edmonds, 2009), the current study reflects the error-prone nature of unfamiliar face identification, with average accuracy across Experiments 1–3 of 66–68% for face matching and 66–69% for face memory. These low accuracy rates raise concern for practical settings in which face identification tasks are frequently conducted. For example, border control officers routinely perform face matching when comparing travellers to their passport photograph to establish that they are the same person, and not an identity impostor. In addition, police officers frequently rely on the accurate face memory ability of eyewitnesses. Recent work has suggested that identification errors in these contexts could be reduced through selecting individuals who demonstrate extraordinary face-recognition ability (e.g., Bindemann, Avetisyan, & Rakow, 2012; Bobak, Dowsett, & Bate, 2016; Robertson, Middleton, & Burton, 2015; Robertson et al., 2016) or by measuring the face recognition ability of eyewitnesses via post-decision tests of identification accuracy (Bindemann, Brown, et al., 2012). The current experiments suggest that when developing a battery of tests to measure general face processing ability in such contexts, a measure of face detection performance would be of limited value.

## Conclusions

In summary, the current experiments provide little evidence to suggest that individual differences in face detection are related to those in face matching and face memory. Thus, the ability to reliably detect faces in one’s visual field appears to be largely unrelated to the subsequent identity processes that this task should, in theory, facilitate. Moreover, although face memory and face matching sometimes engage similar mechanisms, a large proportion of the variability between these tasks remains unaccounted for, indicating that these processes can also operate independently of each other.

## Abbreviations

CFMT(+): Cambridge Face Memory Test (long form)
GFMT: Glasgow Face Matching Test
KFMT: Kent Face Matching Test

## Footnotes

1 For completeness, the combined samples of experiments 2 and 3 were also analysed to investigate whether there was any additional association, with *N* = 100. The results of this analysis did not differ from the findings of experiment 3, except that response times in the detection task also now correlated with response times on match trials and mismatch trials, and with overall correct response times on the KFMT. In addition, all significant results remained significant after Benjamini-Hochberg adjustment.

## References

[CR1] Benjamini Y, Hochberg Y (1995). Controlling the false discovery rate: a practical and powerful approach to multiple testing. Journal of the Royal Statistical Society Series B (Methodological).

[CR2] Bindemann M, Avetisyan M, Rakow T (2012). Who can recognize unfamiliar faces? Individual differences and observer consistency in person identification. Journal of Experimental Psychology: Applied.

[CR3] Bindemann M, Brown C, Koyas T, Russ A (2012). Individual differences in face identification postdict eyewitness accuracy. Journal of Applied Research in Memory and Cognition.

[CR4] Bindemann M, Burton AM (2009). The role of color in human face detection. Cognitive Science.

[CR5] Bindemann M, Burton A, Leuthold H, Schweinberger S (2008). Brain potential correlates of face recognition: geometric distortions and the N250r brain response to stimulus repetitions. Psychophysiology.

[CR6] Bindemann M, Lewis MB (2013). Face detection differs from categorization: evidence from visual search in natural scenes. Psychonomic Bulletin & Review.

[CR7] Bobak AK, Dowsett AJ, Bate S (2016). Solving the border control problem: evidence of enhanced face matching in individuals with extraordinary face recognition skills. PLoS One.

[CR8] Bobak AK, Hancock PJ, Bate S (2016). Super-recognisers in action: evidence from face-matching and face memory tasks. Applied Cognitive Psychology.

[CR9] Bobak AK, Parris BA, Gregory NJ, Bennetts RJ, Bate S (2017). Eye-movement strategies in developmental prosopagnosia and “super” face recognition. Quarterly Journal of Experimental Psychology.

[CR10] Burton AM, Bindemann M (2009). The role of view in human face detection. Vision Research.

[CR11] Burton AM, Schweinberger SR, Jenkins R, Kaufmann JM (2015). Arguments against a configural processing account of familiar face recognition. Perspectives on Psychological Science.

[CR12] Burton AM, White D, McNeill A (2010). The Glasgow Face Matching Test. Behavior Research Methods.

[CR13] Churches O, Baron-Cohen S, Ring H (2009). Seeing face-like objects: an event-related potential study. Neuroreport.

[CR14] Crouzet SM, Thorpe SJ (2011). Low-level cues and ultra-fast face detection. Frontiers in Psychology.

[CR15] Dalrymple KA, Corrow S, Yonas A, Duchaine B (2012). Developmental prosopagnosia in childhood. Cognitive Neuropsychology.

[CR16] Dalrymple KA, Duchaine B (2016). Impaired face detection may explain some but not all cases of developmental prosopagnosia. Developmental Science.

[CR17] Duchaine B, Nakayama K (2006). The Cambridge Face Memory Test: results for neurologically intact individuals and an investigation of its validity using inverted face stimuli and prosopagnosic participants. Neuropsychologia.

[CR18] Foreman N (1991). Correlates of performance on the Gollin and Mooney tests of visual closure. The Journal of General Psychology.

[CR19] Fysh MC, Bindemann M (2018). The Kent Face Matching Test. British Journal of Psychology.

[CR20] Garrido L, Duchaine B, Nakayama K (2008). Face detection in normal and prosopagnosic individuals. Journal of Neuropsychology.

[CR21] Hancock PJ, Bruce V, Burton AM (2000). Recognition of unfamiliar faces. Trends in Cognitive Sciences.

[CR22] Henderson JM (2003). Human gaze control during real-world scene perception. Trends in Cognitive Sciences.

[CR23] Herzmann G, Danthiir V, Schacht A, Sommer W, Wilhelm O (2008). Toward a comprehensive test battery for face cognition: assessment of the tasks. Behavior Research Methods.

[CR24] Hole GJ, George PA, Eaves K, Rasek A (2002). Effects of geometric distortions on face-recognition performance. Perception.

[CR25] Johnston RA, Edmonds AJ (2009). Familiar and unfamiliar face recognition: a review. Memory.

[CR26] Lewis M, Edmonds A (2005). Searching for faces in scrambled scenes. Visual Cognition.

[CR27] McCaffery, J. M., Robertson, D. J., Young, A. W., & Burton, A. M. (in press). Individual differences in face identity processing. Cognitive Research: Principles and Implications*.* 10.1186/s41235-018-0112-9 PMC6019420 30009251

[CR28] Megreya AM, Burton AM (2006). Unfamiliar faces are not faces: evidence from a matching task. Memory & Cognition.

[CR29] Mooney CM (1956). Closure with negative after-images under flickering light. Canadian Journal of Psychology/Revue Canadienne de Psychologie.

[CR30] Moulson MC, Balas B, Nelson C, Sinha P (2011). EEG correlates of categorical and graded face perception. Neuropsychologia.

[CR31] Peirce JW (2007). PsychoPy – psychophysics software in python. Journal of Neuroscience Methods.

[CR32] Pongakkasira K, Bindemann M (2015). The shape of the face template: geometric distortions of faces and their detection in natural scenes. Vision Research.

[CR33] Rayner K (1998). Eye movements in reading and information processing: 20 years of research. Psychological Bulletin.

[CR34] Robertson DJ, Jenkins R, Burton AM (2017). Face detection dissociates from face identification. Visual Cognition.

[CR35] Robertson, D., Middleton, R., & Burton, A. M. (2015). From policing to passport control. *Keesing Journal of Documents & Identity*, *46*, 3–8.

[CR36] Robertson DJ, Noyes E, Dowsett AJ, Jenkins R, Burton AM (2016). Face recognition by metropolitan police super-recognisers. PLoS One.

[CR37] Russell R, Duchaine B, Nakayama K (2009). Super-recognizers: people with extraordinary face recognition ability. Psychonomic Bulletin & Review.

[CR38] Schönbrodt FD, Perugini M (2013). At what sample size do correlations stabilize?. Journal of Research in Personality.

[CR39] Takahashi K, Watanabe K (2013). Gaze cueing by pareidolia faces. Perception.

[CR40] Tsao DY, Livingstone MS (2008). Mechanisms of face perception. Annual Review of Neuroscience.

[CR41] Ulrich PI, Wilkinson DT, Ferguson HJ, Smith LJ, Bindemann M, Johnston RA, Schmalzl L (2017). Perceptual and memorial contributions to developmental prosopagnosia. The Quarterly Journal of Experimental Psychology.

[CR42] Verhallen RJ, Bosten JM, Goodbourn PT, Bargary G, Lawrance-Owen AJ, Mollon JD (2014). An online version of the Mooney face test: phenotypic and genetic associations. Neuropsychologia.

[CR43] Verhallen RJ, Bosten JM, Goodbourn PT, Lawrance-Owen AJ, Bargary G, Mollon JD (2017). General and specific factors in the processing of faces. Vision Research.

[CR45] Wilhelm O, Herzmann G, Kunina O, Danthiir V, Schacht A, Sommer W (2010). Individual differences in perceiving and recognizing faces – one element of social cognition. Journal of Personality and Social Psychology.

